# Characterization of Reusable and Recyclable Plastic Bedding Materials for Laboratory Mice

**DOI:** 10.3390/ani15040501

**Published:** 2025-02-10

**Authors:** Dana Matzek, Bastian Popper

**Affiliations:** Biomedical Center, Core Facility Animal Models, Faculty of Medicine, Ludwig-Maximilians-Universität München, Großhaderner Straße 9, 82152 Planegg-Martinsried, Germany

**Keywords:** polycarbonate, polysulfon, synthetic bedding, mus musculus

## Abstract

The scientific community has placed increasing importance on sustainability, leading to the exploration of alternative bedding materials to the widely used aspen wood chips. Bedding plays a critical role in ensuring the wellbeing of animals and the validity of experimental outcomes. Compared to the frequently evaluated biological materials, such as corncobs or spelts, synthetic materials such as plastic granules have been less investigated. We characterized two thermostable plastic materials as an alternative bedding material in C57BL/6J and BALB/c mice. We examined the impact of those bedding alternatives on physiological parameters, behavior, health status, and cage climate in static and digitally ventilated cages as well as the possibility of a reuse cycle after reprocessing. The mouse lines showed different locomotor activity, feeding, and nestbuilding behavior on plastic granules. While ammonia levels were constantly higher in static cages than in ventilated cages, blood parameters were unaltered, and health status was maintained during the reuse procedure. We show the use of plastic granulate as bedding material for mice that has the potential for processing and recycling. Further, we show that the material is accepted differently by the lines in the preference choice test without affecting their health or hygiene status.

## Simple Summary

The study evaluates thermostable plastic granules as a sustainable alternative to conventional bedding materials for the husbandry of laboratory mice. Physiological parameters and cage climate were assessed in two commonly used mouse strains. The findings revealed different behavioral preferences between these two mouse strains without any negative impact on their health. Additionally, the study demonstrated the successful reusability of plastic bedding after reprocessing, highlighting its potential to improve sustainability in laboratory animal housing.

## 1. Introduction

Bedding material in laboratory mouse cages plays a critical role in ensuring the wellbeing of the animals and the validity of experimental outcomes. Bedding substrates must fulfill multiple roles: absorbing moisture, mitigating odor, and promoting the animals’ comfort and natural behaviors, which are crucial for their physiological and psychological health [1]. Insufficient or inappropriate bedding can lead to increased stress levels in the mice, potentially affecting experimental results by introducing variability in physiological parameters such as body temperature and immune responses [2,3]. Research suggests that enriching the cage environment with adequate materials for burrowing and nestbuilding enhances animal welfare without compromising the consistency of experimental data [4] and bedding volume and material type impact the mice’s ability to maintain normal behavior and thermoregulation [2,5]. In addition to the use of appropriate materials to design the cage microenvironment, the caging system and cage management itself play a decisive role in terms of animal welfare in mice and rats, as they can impact the ammonia concentration [6,7,8,9,10]. The most commonly used bedding materials for laboratory mice are wood-based products, such as aspen shavings, paper-based materials, and corncob bedding. Each of these materials has been extensively studied for its impact on animal welfare, cage climate, and experimental results [7,11,12,13,14,15,16]. Wood shavings are widely used due to their availability and cost-effectiveness, but they can generate dust and volatile compounds that may cause health issues in mice and humans [17,18].

In recent years, the scientific community has placed increasing emphasis on sustainability, leading to research into alternative bedding materials such as corncob and spelt [1]. These substrates offer environmental benefits because they are derived from renewable agriculture. Corncob bedding is valued for its low dust levels and high absorbency, which can further reduce ammonia concentrations in the cage [15,19]. However, it has been shown to have drawbacks, such as the potential to release harmful substances, including aflatoxins and estrogens [20,21]. Further, corncob bedding can impact metabolic studies in which glucose levels are of importance [22]. Further, its hardness can make it uncomfortable for animals [1]. Spelt, on the other hand, is soft and may promote nesting behavior, although concerns have been raised about its palatability, as it may be consumed by the animals and thus interfere with experimental diets [1]. Recycled paper and cellulose-based products are gaining attention due to their sustainability potential, soft characteristics, and better ammonia control compared to corncob in individually ventilated cages [21], which impacts the general wellbeing of mice [23]. However, when choosing the bedding, it must also be kept in mind that it is eaten at least in part or, especially in experiments with food deprivation, as a feed substitute. In these cases, corncob and cellulose products have the disadvantage of changing the intestinal microbial flora [24]. Despite the aforementioned benefits of biodegradable substrates in animal housing, one aspect that always comes to the fore is the proper processing of bedding materials to ensure that they are free of contaminants, such as dust, pathogens, or unwanted chemicals, that could otherwise affect both animal welfare and the outcomes of experiments. Autoclaving or irradiation is often used to ensure pathogen-free bedding, especially for immunocompromised mice or studies that require a controlled environment to avoid microbial interference. However, these methods can alter the physical properties of the bedding, such as its absorbency and structure, which may in turn affect the microenvironmental climate and/or animal comfort [15]. Although the sustainability of the individual products mentioned is outstanding, all of these bedding materials have the disadvantage that they cannot be reused without permanently changing the materials properties. To overcome those limitations, hydrothermal processing has been tested to reprocess bedding material made of softwood spruce in a laboratory environment [25]. However, the bedding was not allowed to contain any contamination of nesting material, which is not feasible under routine laboratory animal husbandry conditions for animal welfare reasons.

Plastic or synthetic bedding, while less commonly used than traditional wood or paper-based materials, has been explored in recent years [26]. The plastic polymer is primarily responsible for its stability. However, depending on the chemicals used, the plastic can have hazardous properties [27]. One significant advantage of plastic bedding is its durability, uniformity, and reuse potential, making it an attractive option for reducing waste in research facilities. Further, plastic bedding materials can be a useful alternative to conventional bedding materials, especially if it is to be expected that wood or biological alternatives could have an impact on the research experiments.

The key questions that need to be answered when using recycled plastic are: How can sterility be maintained during the reuse process? Does it affect animal health and welfare? The aim of the present study was to investigate the suitability of plastic granules, which are normally used for the construction of cage material, as bedding material in a preference choice trial. We made use of two common mouse strains to record spontaneous behavior as well as cage climate parameters. In addition, we have developed and evaluated an internal reprocessing process for soiled plastic bedding material, which allows for the reuse of bedding material in-house and further recycling without posing a hygiene risk factor to mice, and in terms of general biosafety.

## 2. Materials and Methods

### 2.1. Animals

The housing of laboratory mice was in accordance with European and German animal welfare legislations (5.1–231 5682/LMU/BMC/CAM). Five-week-old female BALB/cAnNCrl (cohort 1, *n* = 18) mice (Charles River, Sulzfeld, Germany), six-week-old male (*n* = 6) and female (*n* = 6) C57BL/6JRj mice (Janvier, Le Genest-Saint-Isle, France) of cohorts 2 and 3, and eight-week-old (*n* = 12) female C57BL/6JRj mice (Janvier, Le Genest-Saint-Isle, France) of cohort 4 were either housed in individually ventilated (green line, type II long, Tecniplast, Buguggiate, Italy) or filter top cages (Type II long, Tecniplast, Buguggiate, Italy) in groups of 3–6 individuals. The sample size (animal numbers) is based on the average occupancy densities for type II cages in our facility (*n* = 3 per cage). Animals were randomly allocated to experimental groups. In the preference choice experiments, the groups of male and female animals were divided into two groups of 6 animals each and examined separately (cohorts 1, 2, and 3). Cohort 4 was divided into 4 groups of 3 animals each in each cage.

All mice were housed under specified pathogen-free conditions [28]. Room temperature and relative humidity ranged from 20 to 22 °C and 45 to 55%. The light intensity in the room was set to a maximum of 270 lux. The light intensity inside the cages ranged from 6.1 to 25.3 lux and was monitored by the use of an ST 8820 Environment Meter (ELV Electronic, Leer, Germany). Temperature and relative humidity were recorded continuously in the housing room. The light cycle was adjusted to a 12 h light/12 h dark period. Room air was exchanged 11 times per hour and filtered with HEPA systems. Hygiene monitoring of the facility was performed every three months based on the recommendations of the FELASA-14 working group [28]. All animals had free access to water and food (irradiated, 10 mm pellet; 1314P, Altromin, Laage, Germany). The cages were equipped with nesting material (5 × 5 cm, Nestlet, Datesand, UK), a red corner house (Tecniplast, Buguggiate, Italy), and a rodent play tunnel (7.5 × 3.0 cm, Datesand, UK). Based on the study, the animals had either access to wood bedding material (Animal bedding fine, LTE-E-002, Abedd, Vienna, Austria) or plastic granulate (PC granulate, 3010111, Zoonlab GmbH, Castrop-Rauxel, Germany). The bedding height was set at 2 cm so that natural behaviors such as digging could be practiced. The height corresponds to 240 g wood chips and 920 g PC granules. Soiled bedding was replaced every 7 days. Polysulfon granules (PSU, Tecniplast, Buguggiate, Italy) were not tested in preference choice tests. The cage was changed once a week by the same person. The animals were transferred to a fresh cage under a transfer bench (Aria, CS60, Tecniplast, Buguggiate, Italy) using cup handling.

### 2.2. Characterization of the Plastic Granules

To determine the product features of the two synthetic substrates tested, polycarbonate (PC, 3–4 mm × 2–3 mm Zoonlab GmbH, Castrop-Rauxel, Germany) and polysulfon (PSU, 3–4 mm × 2–3 mm, Tecniplast, Buguggiate, Italy), representative images of the granules were first taken using a stereo microscope connected to a digital camera system (Stemi 508, Carl Zeiss AG, Jena, Germany) equipped with a digital camera (Axiocam 105, Carl Zeiss AG, Jena, Germany). The surface textures were examined and compared in terms of their properties. The longitudinal and transverse axes of 50 randomly selected granules were measured using a digital electronic caliper (FST, Heidelberg, Germany). The granules were also examined with regard to their heat absorption capacity. The cages were littered in the same way as they are offered to the animals, which corresponds to 240 g aspen wood chips, 920 g PC, and 870 g PSU. A heating plate (UNO rodent surgical monitor, UNO life science solutions, Zevenaar, The Netherlands) was placed under a cage and heated to a maximum of 40 °C. The substrate was heated over a period of 60 min. The thermal probe of the monitor system was located in the bedding 1.8 cm upwards of the cage bottom. Recording of the temperature was carried out at 15 min intervals. After 60 min, the heating was stopped, and the cooling behavior was examined for a further 60 min. Thermal images of the surface of the litter were taken during the test using a digital camera (P2 Pro, InfiRay Technologies Co., Hefei, Anhui, China). To test the product’s stability after multiple uses by autoclaving, a random sample of granules from the PC and PSU batch was autoclaved 10 times, and the surface texture was examined using a stereomicroscope (Stemi 508, Carl Zeiss AG, Jena, Germany).

### 2.3. Preference Choice Setup

To investigate the preference for bedding material, mice were housed in digitally ventilate cages (DVC, GM500, Greenline, Tecniplast, Buguggiate, Italy) in two groups of six individuals (cohort 2 and 3) or in filter top cages (FTC, Tecniplast, Buguggiate, Italy) in a group of six individuals (cohort 3). Directly after transport, all mice were transferred into the preference choice setup for acclimatization. The setup consists of two cages (each floor size 501 cm^2^) connected with a transparent tube. The maximal floor size of the setup is indicated to be 1002 cm^2^. During the first two weeks of the preference baseline recording in the DVC system, mice were housed in the modified two-cage interlink system (Tecniplast, Buguggiate, Italy) on aspen wood chip bedding and were allowed to freely explore the entire arena. This period was used for acclimatization and the recording of baseline spontaneous motor and explorative activity. Mice had free access to water and food, and the consumption was monitored on a weekly basis. To ensure that no side preferences due to the cage position in the rack could distort the results, the positions of the cages in the runs of cohorts 2 and 3 were swapped so that in each run one cage was in the middle of the rack and another on the side of the rack. After two weeks of accommodation, polycarbonate (PC) granulate was presented in one of the cages, while the other bedding material presented was still based on aspen wood chips. The enrichment items were not removed during the tests. Only fresh nestlets and play tunnels were presented during routine cage changes. After two weeks of baseline recording, the preference choice test DVC started over a period of six weeks. Body weight was measured by the use of a precision scale (LS1200, G&G GmbH, Kaarst, Germany) once per week during the cage-changing procedures. Spontaneous motor activity in the DVC housing system was analyzed by the use of the Analytics software (V.2019, Tecniplast, Buguggiate, Italy). In FTC, side preference was recorded by quantifying the number of animals in cohort 1 up to 4 times during the light phase. In addition, it was also recorded whether water or food was consumed on either aspen wood bedding or PC granules.

### 2.4. Health Monitoring, Health Check, and Blood Sampling

All mice were subjected to a health monitoring control on the day of delivery, after 7 days, and on day 56. The Interceptor filter paper method (Tecniplast, Buguggiate, Italy) was used to monitor the viral, bacterial, and parasite load of the digital ventilated cages during individual experiments. All pathogens recommended by FELASA for quarterly and annual testing were recorded, and the range of pathogens was also extended to include opportunists [28]. The filter paper was placed inside the dust filter component and inside the exhausted air dust pipe. All samples were evaluated according to the FELASA recommendations from an external company (mfd Diagnostics GmbH, Wendelsheim, Germany), and the health certificate was created. During the routine cage change once per week, all mice were subjected to a health check based on clear criteria (Table S2). At the start and end point of the preference choice tests, 50 µm blood samples were taken from the tail veins and analyzed using the automated Element HT5 (Scilvet, Viernheim, Germany) system as part of the health monitoring concept. The blood glucose values were evaluated with the help of a Sprint nG-1 m (smartLab, Heddesheim, Germany).

### 2.5. Cage and Room Climate Recording

During the experiments, data loggers (Ebro EBI 20-TH-1, Xylem Analytics, Ingolstadt, Germany) were used to measure the temperature and relative humidity inside the room and cages. Ammonia concentration inside the cages was monitored through the use of filter paper tires (AM-40 Hydrion Ammoniak Meter, Micro Essential LAB, Micro Essential Laboratory Inc., New York, NY, USA). The filter strips were inserted through existing openings in the side of the cage without opening the cage and left there for 10 s before the color scale was read. The ammonia concentrations were recorded according to the manufacturer’s protocol in the range of 0, 5, 10, 20, 50, and 100 ppm. Additionally, data loggers placed under the cage lid (Wi-Com, Allentown Inc., Allentown, PA, USA) were used to confirm the results obtained by the filter paper method. The ammonia concentrations were recorded in three stages: 0–20 ppm, 20–30 ppm, and 30–50 ppm.

### 2.6. Body Temperature Monitoring

To record the core body temperature, transponders were used (UNO Temp Micro ID/12 ISP, UNO life science solutions, Zevenaar, The Netherlands), which were implanted subcutaneously in two animals per group. The data were recorded every third day (TR-232128, RFID ISO reader, UNO life science solutions, Zevenaar, The Netherlands). The mean values were calculated from 5 measurements per day (range 5.30 am–8.30 pm).

### 2.7. Eye Examination

At the end of the run of cohort 4, six animals from the group housed on aspen wood chips and 6 animals from the group on PC granules were examined using a slit lamp (Kowa SL19, Tokyo, Japan) at 16× magnification. The animals were killed by cervical dislocation and examined immediately. A total of 24 eyes were evaluated for corneal lesions using the fluorescein test (Opthoresicein 5 mg/mL EDO, CP-Pharma, Burgdorf, Germany), and images were taken with a stereomicroscope (Stemi 508, Carl Zeiss AG, Jena, Germany) equipped with a digital camera (Axiocam 105, Carl Zeiss AG, Jena, Germany).

### 2.8. Bedding Processing

Soiled PC litter was collected for processing and poured onto a self-built vibrating plate with a sieve insert (2 mm). For occupational safety reasons, this vibrating plate was installed in a discharge station with a light source and workplace extraction (Aria DS One, Tecniplast, Buguggiate, Italy). Dirt particles were retained and disposed of during this process. PC granulate was then washed two times in wash bags (Wenko 3 kg, Wenko Wenselar, Hilgen, Germany) at 95 °C and 1400 units per minute (U/min) with the use of 50 mL disinfectant powder detergent (Arenas-Perla, Fa. Kiehl, Odelzhausen, Germany) in a washing machine (PW 5064 Miele Professional, Miele, Gütersloh, Germany). After that, PC granules were allowed to air dry before autoclaving at 121 °C and 2000 mbar (DSL-18, Holzner, Nußlach, Germany). A representative sample of 25 kg of processed PC granules was sent to a company for recycling purposes. The cleanliness of the material was assessed, and the possibilities for further processing were examined.

### 2.9. Statistics

Statistics were conducted using Prism GraphPad 5.04 (GraphPad Software, San Diego, CA, USA). Student’s *t*-test was applied for parametric data. A Kruskal–Wallis (K-W) test followed by Dunn’s multiple comparison (non-parametric data) was used to determine the *p*-values. Data are shown as mean and SEM values if not stated otherwise. The significance level was set to *α* < 0.05.

## 3. Results

**Figure 1:**
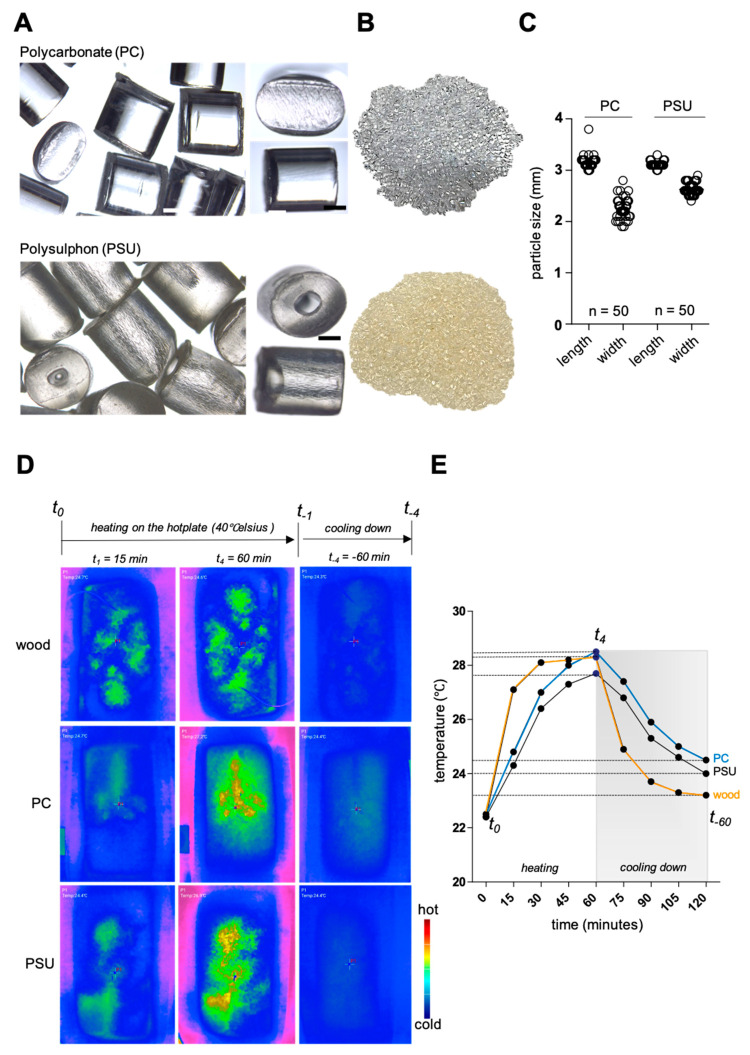
(**A**) Representative detailed images of polycarbonate (PC) and polysulfon (PSU) plastic granules. (**B**) Representative images to visualize color differences. (**C**) Determination of the particle dimensions. (**D**) Selected thermal images of the plastic bedding in the cage during the 60 min heating and cooling phase. (**E**) Graphical recording of all measurement data for PC, PSU, and aspen wood chips. *n* = number of particles. t_0–1–4_ = quarter-hourly measurement times of the heating phase, t_−1–4_ = quarter-hourly measurement times of the cooling phase. Scale bar = 1 mm.

We first investigated the basic properties of the two synthetic bedding materials for which we wanted to evaluate their impact on animal welfare: polycarbonate (PC) and polysulfon (PSU). To this end, we evaluated the morphology of the granules (Figure 1A). The surface of the PC granules is smooth and shiny, with small edges and small indentations on the cut surfaces and sides of the cylindrical and round-oval particles. PSU granules have a shiny surface on the cylindrical particles, which are not smooth but have fine lines and cracks on the side surface (Figure 1A). On the cut surface, the granules usually have a round-oval cavity, which sometimes runs like a tube through the entire cylinder, or at least partially through it. The color of the two granules differs. While PC granules have a whitish, almost lustrous character, PSU granules appear matte yellowish (Figure 1B). Both granules are not significantly different in terms of particle size. A slight trend toward narrower, but not shorter, PC granules compared to PSU was observed (Figure 1C).

As the materials are thermostable, their heat storage capacity was investigated (Figure 1D). Thermal images of the bedding were taken and evaluated in parallel (Figure 1D). During the heating phase, a nest-like warming can be observed in aspen wood chips, predominantly in the middle of the cage, and a steep rise in temperature. PC and PSU granules showed homogeneous heating, even if this did not cover all areas of the cage floor evenly. The rise in the heat curve was less steep than with aspen wood chips (Figure 1E). Even after 60 min, no homogeneous distribution of heat could be seen in the picture with aspen wood chip bedding (Figure 1D). Large portions of the central cage area that were covered with PC and PSU granules were heated up to around 28 °C after 60 min. The highest temperature was recorded for PC granules. After the heating phase, the cooling behavior of the materials was recorded. While aspen wood chips lost heat very quickly, PC and PSU granules stored the temperature and differed from aspen wood chips by around 1 °C at the end of the 60 min cooling phase (Figure 1E). Based on these preliminary tests and the described material properties, e.g., temperature storage, we decided to only use PC granules for the behavioral tests.

**Figure 2:**
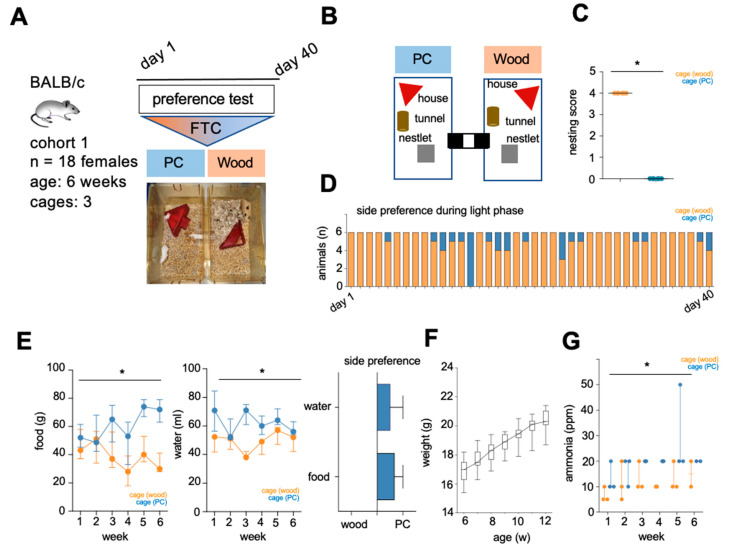
(**A**) Workflow of preference test of cohort 1 in filter top cages (FTC). (**B**) Graphic design to visualize the enrichment and intra-cage connection. (**C**) Recording of nesting quality on aspen wood chips and plastic bedding at each cage change. (**D**) Recording the average number of animals per cage side at several points throughout the light phase. (**E**) Quantification of food and water intake per cage as well as side preference (arbitrary unit). (**F**) Determination of body weights over the test period. (**G**) Ammonia measurement on the respective cage sides with the aid of filter strips after 7 days of use. Data are presented as mean and SEM (**E**) or median and range (**F**,**G**); significance level p * < 0.05, *n* = number of animals, w = weeks.

In order to assess the influence of synthetic bedding materials on animal behavior, female BALB/c mice were first subjected to a choice experiment in stationary filter-top cages (FTC) (Figure 2A). The enrichment of the cages was identical (Figure 2B). With regard to nestbuilding behavior [29], the animals preferred the aspen wood chip bedding without exception and built nests that sometimes exceeded the height of the animals (Figure 2C). When assessing the lateral preference, the animals were more often found in the cage bedded with aspen wood chips during the light phase (Figure 2D). In terms of food and water intake, however, the animals drank and ate significantly more on the side filled with PC granules compared to aspen wood chips. This was also confirmed by recording the frequency of the basic needs performed during the day phase and evaluated using the side preference (Figure 2E). All examined animals of this cohort showed a line-typical weight development in this test setup (Figure 2F). The ammonia concentration was determined during the six-week test phase using the filter strip method every 7 days, shortly before the change of bedding material. Cages with PC granulate bedding showed a significant increase in ammonia concentration (mean 20 ppm PC vs. 11.67 ppm aspen wood chips) compared to aspen wood chip bedding (Figure 2G).

**Figure 3:**
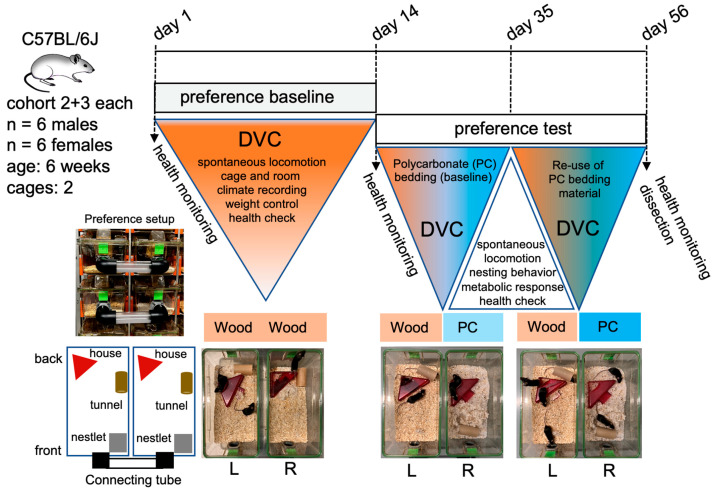
Workflow and representative images of preference test setup of cohort 2 and 3 in digital ventilated cages (DVC). A baseline survey of behavioral data and cage climate data was carried out over 14 days. The cage sides in the choice experiment L (left) and R (right) were provided with the same amount of aspen wood chip bedding. After that, the preference test phase started by replacing the bedding on the R side with polycarbonate (PC) plastic granules. On day 35, the animals were presented with reprocessed bedding on the R side. After 56 days, the animals were dissected, and health monitoring was performed.

To assess the use of cages with PC granules over a period of 6 weeks, including the dark phase, the DVC housing system was employed in the next setup (Figure 3). In this preference test, both sexes of C57BL/6J mice were studied in individually ventilated cages. These cages featured a tube mounted at the front, providing the animals with a choice between aspen wood chip bedding and PC granules (Figure 3). To allow the animals to familiarize themselves with the test setup, mice were initially housed in cages with aspen wood chip bedding in both cages. The six animals per cage and gender were observed over a 14-day period to determine baseline values for animal and environmental parameters. Then, in one cage, the aspen wood chip bedding was replaced by PC granules for a period of 6 weeks. The overall 12 animals were divided into two cohorts, each represented by 6 animals (cohorts 2 and 3) that are either males or females.

**Figure 4:**
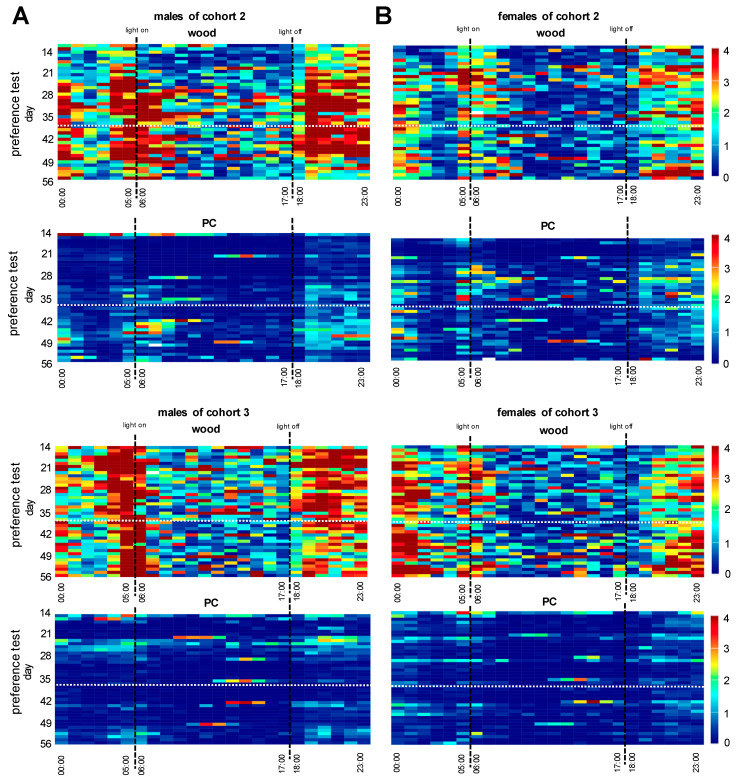
(**A**) Heatmaps of the spontaneous locomotor activity recorded in males. (**B**) Heatmaps of the spontaneous locomotor activity recorded in females. Date is presented over 24 h and 56 days in the DVC system. The animal groups were examined in two sub-sets and were evaluated separately in cohort 2 (*n* = 6 males and *n* = 6 females) and cohort 3 (*n* = 6 males and *n* = 6 females). The activity patterns are shown separately for the cages with aspen wood bedding and polycarbonate (PC) bedding. The light switching times are indicated by black dashed lines. White dashed lines mark the changeover from first-time-used plastic bedding to reprocessed bedding.

Heatmaps were generated from the animal’s spontaneous locomotor behavior for both cohorts during the baseline phase (Figure S1). We could not identify differences in locomotor activities of males or females in the two cohorts tested, while females tend to be less active compared to males. For both sexes, the most activity was recorded during the dark phase. After two weeks of baseline recording, spontaneous locomotor activity was monitored for an additional 6 weeks in the preference test as depicted in Figure 3. Males of cohorts 2 and 3 showed higher locomotor activity in the dark phase and a little less during the light phase in cages equipped with aspen wood chips, while cages with PC granules were less used during the day (Figure 4A). Similar results were obtained from both female cohorts, which showed overall less activity compared to males (Figure 4B).

**Figure 5:**
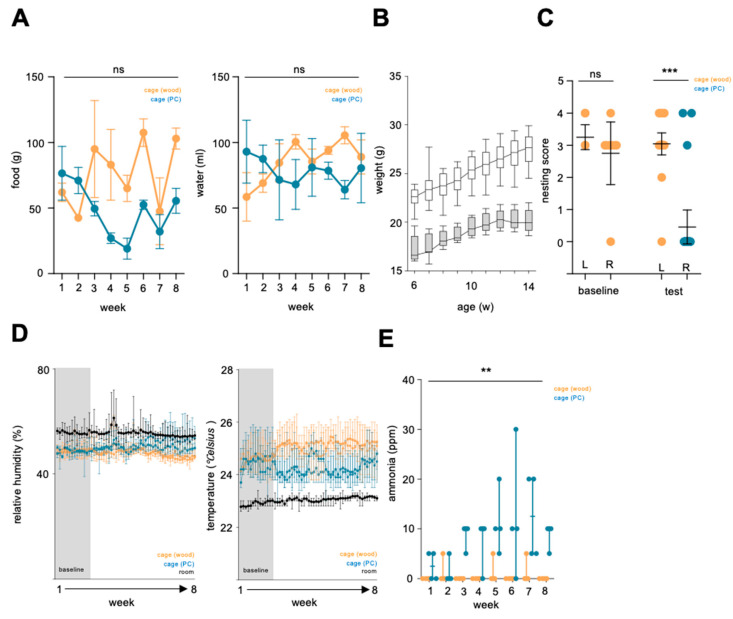
(**A**) Food and water intake of cohort 2 and 3 in digital ventilated cages (DVC) on either aspen wood chip bedding or polycarbonate (PC) granules. (**B**) Growth chart of males (white) females (grey) along the test phase. (**C**) Evaluation of nestbuilding behavior during the baseline and test phase at the time of cage change. (**D**) Recording of relative humidity and temperature inside the cages during the preference baseline and test phase. (**E**) Measurement of ammonia concentration in cages equipped with either aspen wood or PC granules. Data are presented as mean and SEM (**A**,**C**,**D**) or median and range (**B**,**E**), significance level *p* < 0.05, ** = *p* < 0.01, *** = *p* < 0.001, ns = not significant, w = weeks.

Food and water intake was monitored along the cohorts during baseline and test phases, showing lower food and water intake of C57BL/6J on PC granules compared to aspen wood chips (Figure 5A). Body weight gain was not altered in both sexes tested during the preference test (Figure 5B), while nesting behavior was significantly altered in cages having PC granules compared to aspen wood chip bedding. During the baseline phase, both sexes built nests on both sides of the preference cage setup (Figure 5C). Cage climate recording revealed a drop in cage temperature after the baseline phase, indicating less use of the cages with synthetic granules compared to aspen wood chip bedding, while the relative humidity was not changed (Figure 5D). Ammonia levels inside the cages, measured on the day of routine cage changing, were significantly increased in cages with PC granules (mean 8.12 ppm PC vs. 0.46 ppm aspen wood chip bedding). A single measurement point reached 30 ppm in the DVC preference test setup (Figure 5E).

**Figure 6:**
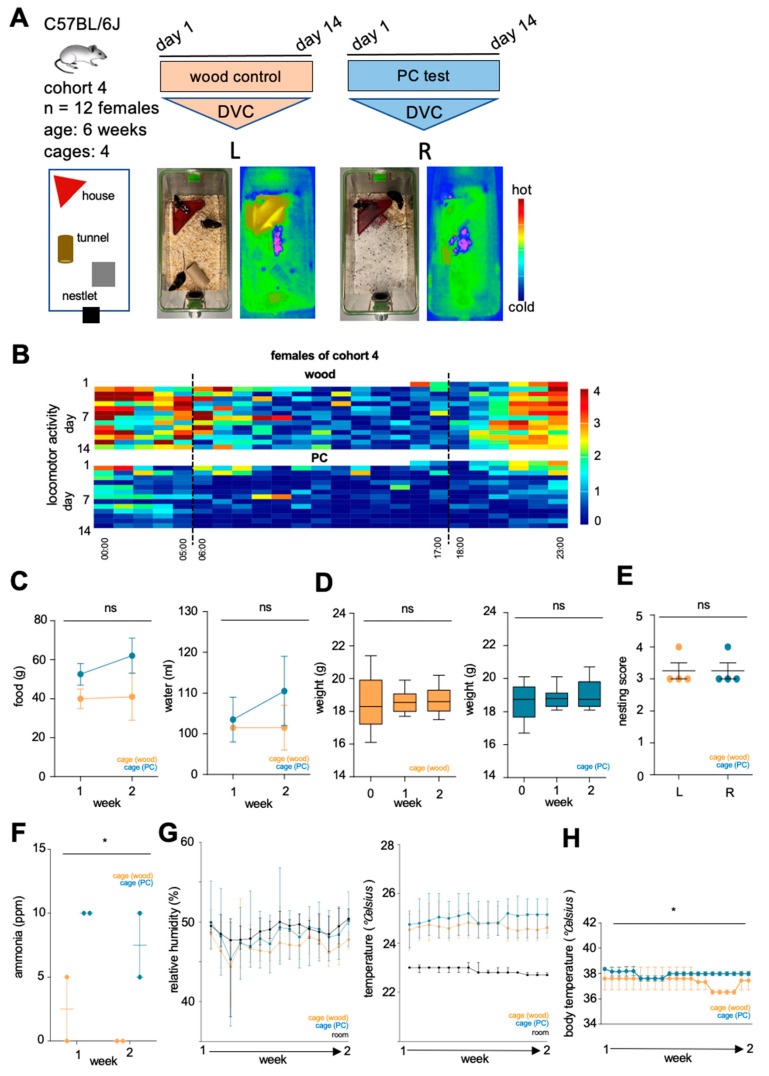
(**A**) Workflow: graphical imaging of the cage design as well as representative thermographic images of used aspen wood chip and polycarbonate (PC) bedding. (**B**) Heatmaps of the spontaneous locomotor activity of cohort 4 (*n* = 12 females) during the 14-day trial on aspen wood chips or PC granules in digital ventilated cages (DVC). (**C**) Food and water intake of cohort 4. (**D**) Growth chart on either aspen wood chip bedding or plastic bedding. (**E**) Evaluation of nestbuilding behavior. (**F**) Measurement of ammonia concentration in cages equipped with either aspen wood chips or PC granules. (**G**) Recording of relative humidity and temperature inside the cages. (**H**) Measurement of body temperature along the test phase (*n* = 4). Data are presented as mean and SEM (**C**,**E**–**H**) or median and range (**D**), significance level * p < 0.05, ns = not significant, L = left side, R = right side.

To further validate the synthetic bedding material, a cohort of 12 C57BL/6J females were housed in DVC cages either on aspen wood chip bedding or on PC granules for 14 days (Figure 6A). Representative thermographic images of cages taken at the time of routine cage changing on day 7 showed a homogeneous bedding temperature and urine and fecal areas along the cage ends behind the house indicated by a lower thermographic signal (Figure 6A). Spontaneous locomotor activity was lower in females housed exclusively on PC granules compared to mice housed on aspen wood chips (Figure 6B). Feeding behavior and water consumption were not altered in those mice, and weight gain was similar in both groups investigated (Figure 6C,D). Further, nesting scores are identical among the 6 mice tested on either aspen wood chips or PC bedding (Figure 6E). The cage ammonia levels were higher in the cages of the PC cohort, reaching a maximum of 10 ppm (mean 8.75 ppm PC vs. 1.25 ppm aspen wood chip bedding) (Figure 6F), while the temperature and relative humidity in both cage conditions were not altered (Figure 6G). To investigate the effect of PC granules on animal body temperature, we used subcutaneously injected transponders to monitor the animals’ body temperatures continuously. The mean values of five measurements per day for the test period showed differences between animal body temperatures in the two test setups (Figure 6H).

**Figure 7:**
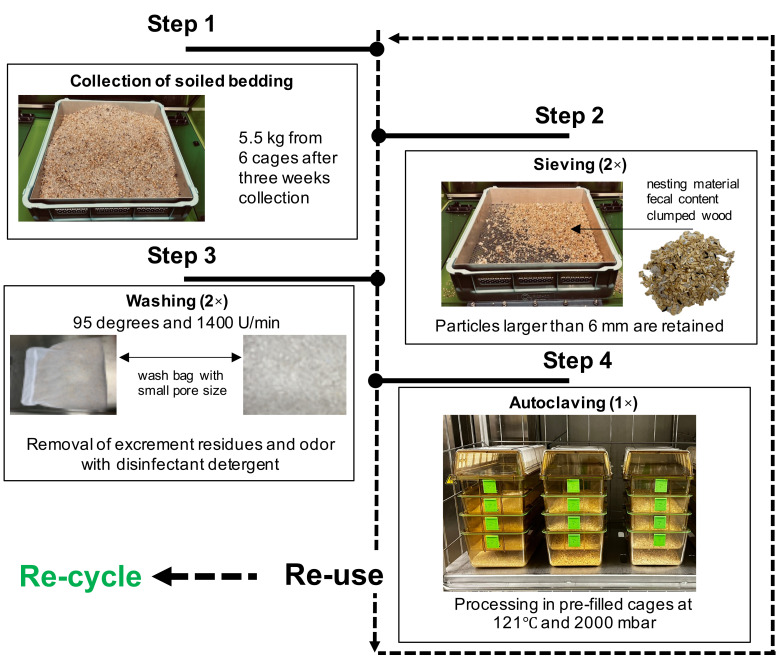
Workflow and representative images of the four-stage reprocessing cascade of soiled PC granules. In addition to reuse after step 4, the recycling option after complete decontamination is also shown in the diagram.

All animals of cohorts 2, 3, and 4 underwent a health examination at the beginning and end of the study. The blood parameters recorded in cohorts 2 and 3 did not differ between male and female mice used in the preference tests (Table S1). Further, female mice of cohort 4 housed on aspen wood chips did not differ in terms of blood parameters from animals housed on synthetic PC granules. Next, because it can be assumed that plastic could lead to injuries if it comes into contact with the eye and because ammonia can have an influence on eye health, we performed slit lamp examinations of the anterior segment of the eye of cohort 4. The corneas did not reveal any fluorescein-positive areas, and both groups showed an intact tear film and no changes in the anterior chamber and lens (Figure S2). Further, no abnormalities were observed in animals housed on either aspen wood chips or PC granules (Table S2). The hygiene status of the housing system and thus also the infection risk to animals were determined using the exhaust air filter method. The examination of two filters at two different locations in the exhaust air duct at the end of the test series showed that the animals were not exposed to any potential mouse pathogens. To determine whether the PC granules we use can be reprocessed to provide reusable bedding for animals, we established a four-step material preparation process (Figure 7).

After the bedding was used and contaminated for 7 days in the trials, an initial quantity of 5.5 kg of the material was collected (step 1). The bedding material was then coarse screened using a sieve (step 2) to retain particles larger than 6 mm, such as aspen wood chip residues, nesting components, and fecal particles that were discarded. The remaining PC granules stuck in nesting material or on fecal matter were disposed of. Subsequently, PC granules were packed into wash bags and cleaned in a washing machine at a high temperature (step 3). Two cycles on a high rotation were run to remove contaminants properly. After that, PC granules were autoclaved at 121 °C and transferred into the barriers for reuse. Complete decontamination of the described process was verified by re-administration of the litter in cohorts 2 and 3, in which animals received processed bedding from day 35 onwards (Figure 3). The re-administration did not change the animals’ locomotor behavior (Figure 4). The blood parameters at the end of the test series were unchanged compared to the blood values at the beginning of the study, and hygiene monitoring using the exhaust air dust method did not result in any changes. Importantly, this autoclaving cycle was repeated 11 times with no detectable changes in particle structure. After reusing the bedding in the livestock, we also checked the particles for their recycling ability (Figure 7). For this purpose, a quantity of 25 kg of the processed bedding was sent to a company to test whether the processed granulate was suitable for further processing. We then determined the use of aspen wood chips compared to plastic in a simple cost calculation, taking into account the loss of PC granulate during the cleaning process and the acquisition costs at the time of our study (Table S3).

## 4. Discussion

The use of sustainable raw materials is of great ecological and socio-political interest. In essence, this means using available resources wisely, protecting the environment, and reducing waste. The use of recyclable raw materials as bedding material in laboratory animal husbandry has therefore attracted the attention of the scientific community in recent years in order to make scientific progress [1]. These recyclable materials should be absorbent, dust-free, non-toxic, and free of pathogens. Moreover, they should meet the needs of the species for which they are used, allowing and encouraging species-specific behaviors such as digging or nestbuilding. This is also recommended by professional organizations and required by law [30]. The commonly used wood shavings and chips from aspen, for example, have been compared with a variety of biodegradable alternatives for decades [12,13,31]. The effects of bedding materials as well as cage change intervals on animals’ physiology and/or cage climate have been studied [8,9,21,32,33,34,35]. In most studies comparing materials and amounts of bedding substrate, different properties of the materials were found to affect the absorbency of the substrates and therefore the ability to absorb liquid excrements [6,32,36]. Particularly in facilities in which individually ventilated cages (IVCs) are absent, odor pollution from high ammonia levels is a significant problem that can affect animal health. In IVC housing systems, the sustainable bedding alternatives of corncob and cellulose-based products appear to be superior to the traditional aspen chips or shavings in terms of ammonia concentration [21]. There are therefore still positive aspects that speak in favor of the use of renewable raw materials as animal bedding rather than wood bedding when using certain caging systems [21,31]. What all studies have in common is that the current limitation is the reuse of the alternative substrates. This may be due to hygiene concerns that limit the use of reusable materials in the hygiene areas of standard laboratory animal facilities, as some natural products have the potential to be contaminated by toxins before first use in the laboratory environment [18,20]. However, it has already been shown that, under certain conditions, the wood reprocessing appears to be technically feasible without negative impacts on animals wellbeing [25].

Based on the concept of sustainable resource utilization, we established an innovative reuse concept for the in-house reprocessing of soiled bedding material. Therefore, we aimed to reuse and recycle synthetic plastic bedding materials instead of renewable raw materials, offering a sustainable approach that does not impact animals’ wellbeing and allows for the maintenance of high hygiene standards. To this end, we characterized two thermostable synthetic plastic granules that are commonly used in the manufacture of animal housing equipment, such as bottles, houses, or cage trays. Polycarbonate (PC) and polysulfon (PSU) materials exhibited different product properties affecting the surface, color, and heat storage capacity. The improved heat storage and distribution of thermostable materials make these synthetic bedding alternatives particularly interesting for animals with increased health requirements, such as nude mice or anesthetized animals [37]. Based on the study by Bellin et al. [26], we specifically used BALB/c females for this initial preference choice screening. In the cited study, polyethylene beads, slightly deviating in size from the PC granules tested, were evaluated in static cages, including an absorbent layer underneath the beads to soak up liquid excrements. Unfortunately, soaking up the urine did not protect the animals from getting wet; the animals in the static cage system were soaked and showed discomfort towards the bedding. The authors discussed that the use of the bedding in a well-ventilated cage system such as the IVC may be better accepted by the animals as the high air exchange is expected to have a drying effect. In contrast to the study by Bellin et al. [26], we did not use any absorbent materials. Interestingly, we did not find any wet animals over the entire study period when using PC granules, but ammonia levels tend to be higher in cages equipped with PC granules. In the behavior experiment, BALB/c mice preferred the cage side with PC granules significantly more often for food and water intake. It was also evident that the animals separated feeding, urination, sleeping, and nestbuilding as soon as they had the opportunity to structure their environment due to the two-cage connection. To counteract the higher ammonia levels in the static preference choice arena, we next evaluated PC granules in IVC cage system. Especially the DVC system, which further allows for 24/7 monitoring of the animals’ spontaneous locomotor activity, was used to test the acceptance of C57BL/6J male and female mice. In line with reports on the behavior of these mouse strains, typical diurnal locomotor activity was recorded in the DVC system [38]. The spontaneous locomotor activity of the animals was reduced on PC granules compared to the baseline data obtained on aspen wood chip bedding. Further, in contrast to the preferred use of the PC granules for food and water intake in BALB/c mice, C57BL/6J mice eat and drink more on wood chip bedding, which reached no statistical significance. C57BL/6J mice of both sexes showed nesting behavior on synthetic bedding but of low quality. Of note, nestbuilding per se differs between laboratory strains and, further, in the need for nestbuilding for thermoregulation purposes. Here, BALB/c mice are more prone to thermal stress compared to C57BL/6J mice [39]. This might be the reason that BALB/c nesting scores are higher compared to C57BL/6J mice among a variety of nesting materials [40]. Another reason why nestbuilding did not work so well in both lines tested may have been the bedding, as both lines prefer deep bedding, especially the C57BL/6J strain [41]. But we found no evidence to suggest that the bedding material impacts nestbuilding on PC granules, since bedding height was identical between the groups tested. Furthermore, a study shows a connection between bedding height and changes in organ weight and blood values [2]. Since we found no difference in the blood values, we conclude that the bedding height had no effect on the animal’s physiology and nestbuilding behavior. The use of the DVC resulted in a drastic reduction in the ammonia concentration compared to the static filter top cages, but the ammonia level remained significantly high compared to the reference side. While values barely exceeding 0 ppm were measured on aspen wood chips, the values from cages with PC granulate were around 10 ppm, reaching a maximum at 30 ppm. The fact that the values increased overall on PC granulate in the course of the experiment may be due to the fact that C57BL/6J mice structured their environment in the preference choice setup so that excrement was increasingly deposited on PC granulate instead of wood. This is also consistent with the findings from the preference choice experiment performed with BALB/c mice. After it became clear that there are mouse line-dependent preferences for the use of PC bedding and that these presumably arise from the structuring of the housing environment, we investigated the impact of PC granules on animal behavior and health in single IVC cages without preference choice abilities. Female C57BL/6J mice were used due to their greater sensitivity to thermal changes [39]. We identified mice structuring the cage for defecation and resting. Urine areas are preferentially located on the cage wall behind the house, which in turn was used as the main resting place in wood chip and PC bedding cages. This finding indicates that cage structure impacts the animals’ behavior independent of the bedding material provided and the size of the enclosure. Like in the preference choice test, female C57BL/6J mice, while showing typical diurnal locomotor activity, are less active on PC granules during the 14 days of testing. Notably, food and water intake, as well as nestbuilding, was as good on PC granules as on aspen wood chips, which led us to the conclusion that synthetic bedding material might have a minor impact on the animals’ wellbeing and physiology. One could argue that less activity leads to lower core body temperatures, and this is compensated by nestbuilding and higher nutrition intake. In our study, core body temperature was higher in mice housed in PC granule-equipped cages, while cage temperature and humidity were not altered during the test phase. We deduce from this that the good thermostable properties of the granulate in terms of heat storage capacity come into play to maintain body temperature, which is further strengthened through nestbuilding and thermotaxis on PC granules, which has been reported as a favored behavior of inactive C57BL/6J mice [42]. Another reason for less spontaneous locomotor activity might be the higher ammonia levels in cages containing PC granules, confirming the lack of absorbent capabilities in those cages. Although the ammonia levels for plastic-lined cages are higher than those for aspen in our study, there are no legal requirements that set maximum limits for ammonia levels in mouse cages, other than those for relative humidity and temperature. Therefore, studies that compared different bedding materials or bedding volumes to set maximum ammonia limits were used to compare our data. Through the use of 14-day cage change intervals using IVC cages, the proposed upper limits of ammonia concentration can be set to 50 ppm (average 22 ppm) [6,21,43]. In all studies, the number of animals, age of animals, and time of day of sampling, as well as the measurement device itself, played a decisive role in recording the ammonia values. Since cages with PC granules did not reach the maximum of 50 ppm, we propose that PC granules cannot be classified as unsuitable per se, and different activity profiles result from the different structuring of the usable cage area. We also investigated whether the granules or the cage climate could have an influence on the health status of the animals. Chronic stress, e.g., mediated by altered nutrition intake, reduced activity, and high ammonia levels that might impact the respiratory system, is typically mediated by alterations of the myeloid cell population [44]. Supporting our hypothesis that cage climate did not alter animal health are the robust blood cell counts and blood glucose levels during the course of experimentation. Contrary to other studies that describe an influence of corncob bedding on metabolic parameters such as glucose [22], PC granules do not appear to have as much potential to alter experimental outcomes. Further, we could also state that the reprocessing of PC granules had no impact on blood cells and metabolic parameters. In particular, absolute white blood cell counts were not excessive in males or females housed on PC granules either in cohorts 2 and 3 or cohort 4, indicating that animals did not suffer from stress or hygiene deficits during the bedding experiments. Similar data were obtained by Miyamoto et al., who investigated the reprocessing of wooden bedding material for the first time [25]. Based on that, one of the central questions of this study was to find out whether soiled synthetic materials can be reprocessed in such a way that they can be reused again without any hygiene risk. In accordance with the blood parameters obtained and the monitoring of the DVC’s exhaust air system, it can be stated that our processing cascade allows for appropriate reprocessing of PC granulate with respect to animal health and biosafety aspects of a genetic engineering facility. Furthermore, our cascade for processing the bedding ensures that odor components are completely removed from the granules. This was confirmed by our recordings of the activity profiles, which did not change after the presentation of prepared litter in the cage. In addition, the processing was so thorough that it was basically possible to recycle the material, which was confirmed by an external company specialized in the recycling of plastic components. In addition to the possibility of recycling the material, we also investigated the possibility of multiple reprocessings. In this case, the particles were autoclaved up to 11 times in addition to the washing cycles. Based on the fact that thermostable granules were tested, we were unable to detect any structural changes in the PC and PSU particles after multiple rounds of processing. Even if reprocessing is technically possible and the mice studied here used the granules to structure their environment in the preference choice experiment, the question will probably arise as to whether the synthetic particles or microplastics can be ingested by the animals and whether this could have a negative impact on animal welfare. Indeed, there are several studies using mice that describe a connection between the accumulation of microplastic in the body and its impact on cell metabolism and body functions [45,46]. Here, we would like to argue that the PC granules used in this study are also used for the production of bottles, houses, and cage trays. The risk of the incorporation of substances should not be higher than after the multiple reprocessing of the mentioned utensils in an animal facility under normal conditions. This means using chemicals for washing and autoclaving multiple times until the end of the product’s lifespan, which exceeds years. We could not identify animals chewing on granules, nor could we find granules in the gastrointestinal tract after dissection. Even if plastics cannot be considered sustainable in comparison to raw materials obtained from agriculture, we believe that in-house processing offers a number of economic advantages, e.g., independently from suppliers, reduced transport costs, and lowered waste disposal, that make plastic bedding an interesting variable in laboratory animal science. Based on our sample calculations, we had high investment costs for the purchase of the granulate (Table S3). However, taking into account the world market prices, the investment costs can be more than halved compared to aspen wood chips if the bedding is reprocessed three times. This highlights the potential for the in-house processing of used bedding material. Indeed, disadvantages might be, e.g., heavier cages compared to aspen wood chips, additional spaces to store granules, additional washing machines, and additional labor.

Our feasibility study has its limitations, and we provide suggestions to overcome those hurdles as follows. We could find mouse line-specific differences in the acceptance of synthetic bedding. While the two mouse lines and genders tested showed reduced spontaneous locomotor activity on PC granules, this might be due to light reflections of the PC granules or the color itself. In fact, there seems to be a correlation between the animals’ preference for the color of the bedding material and the color of their fur [47]. For our study, the C57BL/6J mice would therefore have preferred a darker bedding color. To counter this limitation, opaque PSU plastics, which are also suitable, should be used for further studies. In addition, preference behaviors may have been influenced by housing test animals on aspen bedding before testing. The latter could have had a habitual effect on the animals, so future studies need to evaluate the animal’s behavior in mice reared on plastic granules to rule out any habituation effect. Bedding size and shape might have an impact on animals’ acceptance, as reported from similar experimental studies using C57BL/6J mice, who preferably choose bedding that is not too fine [48]. In this case, other plastic materials that vary in size and shape are available that need to be investigated in further studies. Further, the breeding and rearing behavior must be evaluated before general use can be envisaged. Another limitation is the measurement of body temperature using transponders on only two animals of cohort 4. Even if these differences were shown in the evaluation, the influence of the bedding material on the body temperature as well as the influence of the cycle status in female animals must be ruled out by using a larger number of animals. It must also be ensured that the cage weight does not become a disadvantage for the animal keepers and that the cage weight is equalized to that of wood chips in future studies using plastic bedding material. In relation to optimizing the cage climate, increased air exchange rates, optimized air streams, and/or absorbent materials under the bedding might contribute to the reduction in ammonia levels. We would like to state that synthetic PC bedding can already be used in certain areas of laboratory animal science, particularly with regard to the possibility of hygienic reprocessing and its reuse and recycling aspects. These include, for example, digestibility experiments in which the absorption of excretion products by wood or other materials would have negative effects on the experiment or recovery cages for post-operative situations in which long-lasting heat storage is desired and dust from wood or other materials could influence wound healing. Synthetic bedding could even be a good alternative to conventional bedding materials for keeping gnotobiots, which have special requirements in terms of sterility and microbiome. Further studies, including other strains and species, as well as reprocessing cascades that are economical for large facilities, are necessary to make synthetic bedding available for widespread use. In addition, other raw plastics must also be tested, as well as plastics that themselves come from a recycling process, e.g., material from cages that exceed their lifespan. Only by taking these aspects into account can alternative bedding materials contribute to sustainability in laboratory animal science.

## 5. Conclusions

This study lays the foundation for including plastic cage bedding in the circular economy in laboratory animal husbandry. Based on the concept that plastic bedding can be recycled (e.g., plastic bedding becomes a cage, a cage becomes plastic bedding), it opens up countless possibilities. So far, synthetic materials have only been used to a limited extent for the general housing of laboratory animals, but they can already represent alternatives for certain fields of research and contribute to the robustness of data collection. The results of our study will contribute to a better understanding of the trade-offs between sustainability and animal welfare in laboratory animal science.

## Data Availability

All data are provided in the manuscript.

## Supplementary Materials

The following supporting information can be downloaded at: https://www.mdpi.com/article/10.3390/ani15040501/s1, Figure S1: Heatmaps of spontaneous locomotor activity of cohort 2 and 3 during the baseline phase; Figure S2: Representative images of fluorescein test and quantification of corneal lesions; Table S1: Blood parameters of cohort 2, 3 and 4; Table S2: Criteria used for health checks; Table S3: Example calculation for the use of PC bedding in the in-house reuse and recycle process.
