# Supplementary material for: Characterization of Reusable and Recyclable Plastic Bedding Materials for Laboratory Mice
Source: Animals (Basel). 2025 Feb 10;15(4):501. doi: 10.3390/ani15040501 (PMC11851371; doi:10.3390/ani15040501)
Supplement: Supplementary file 1 [file animals-15-00501-s001.zip › animals-3477767-supplementary.pptx]

## Slide 1
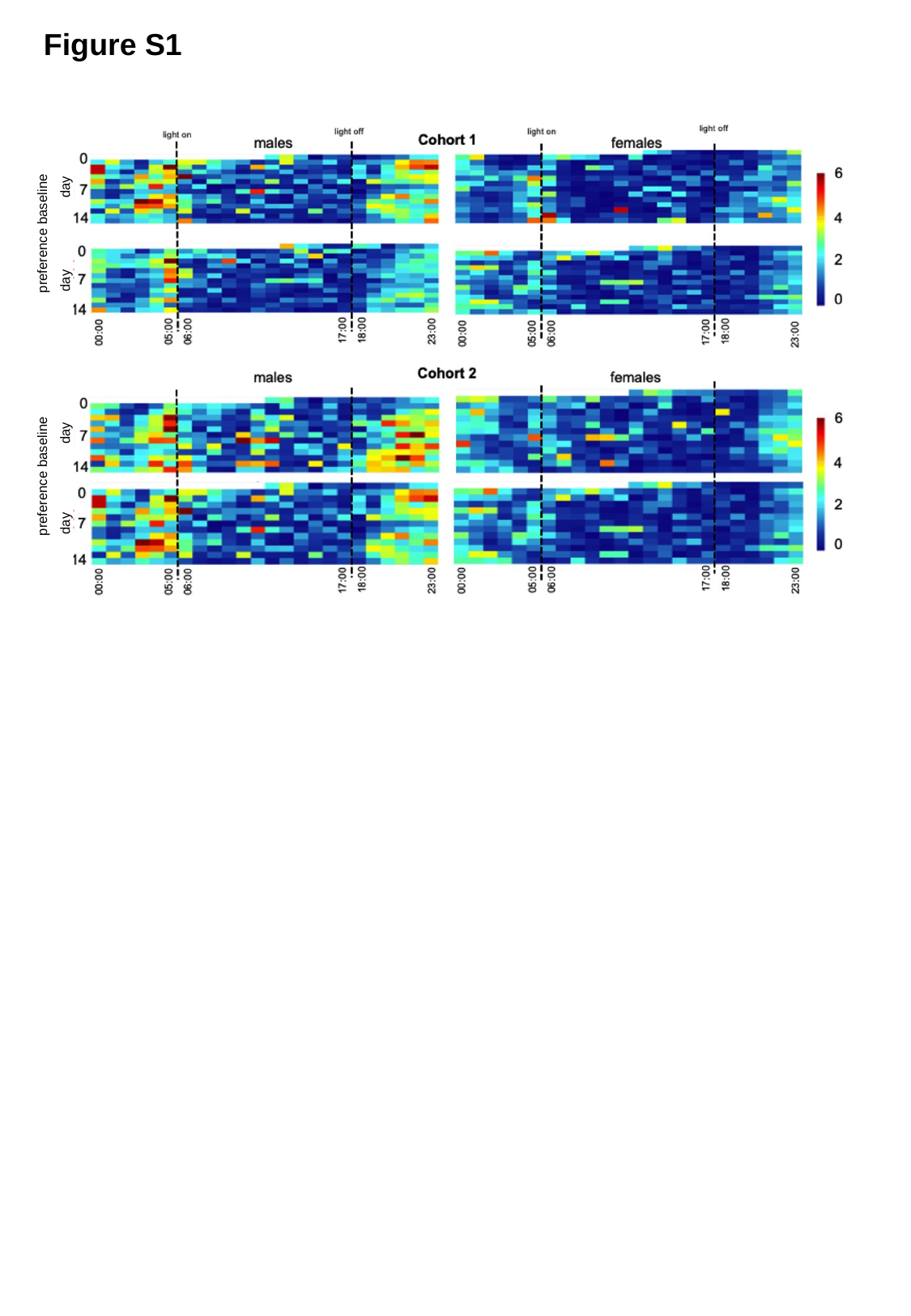

Figure S1
day
preference baseline
day
day
preference baseline
day

## Slide 2
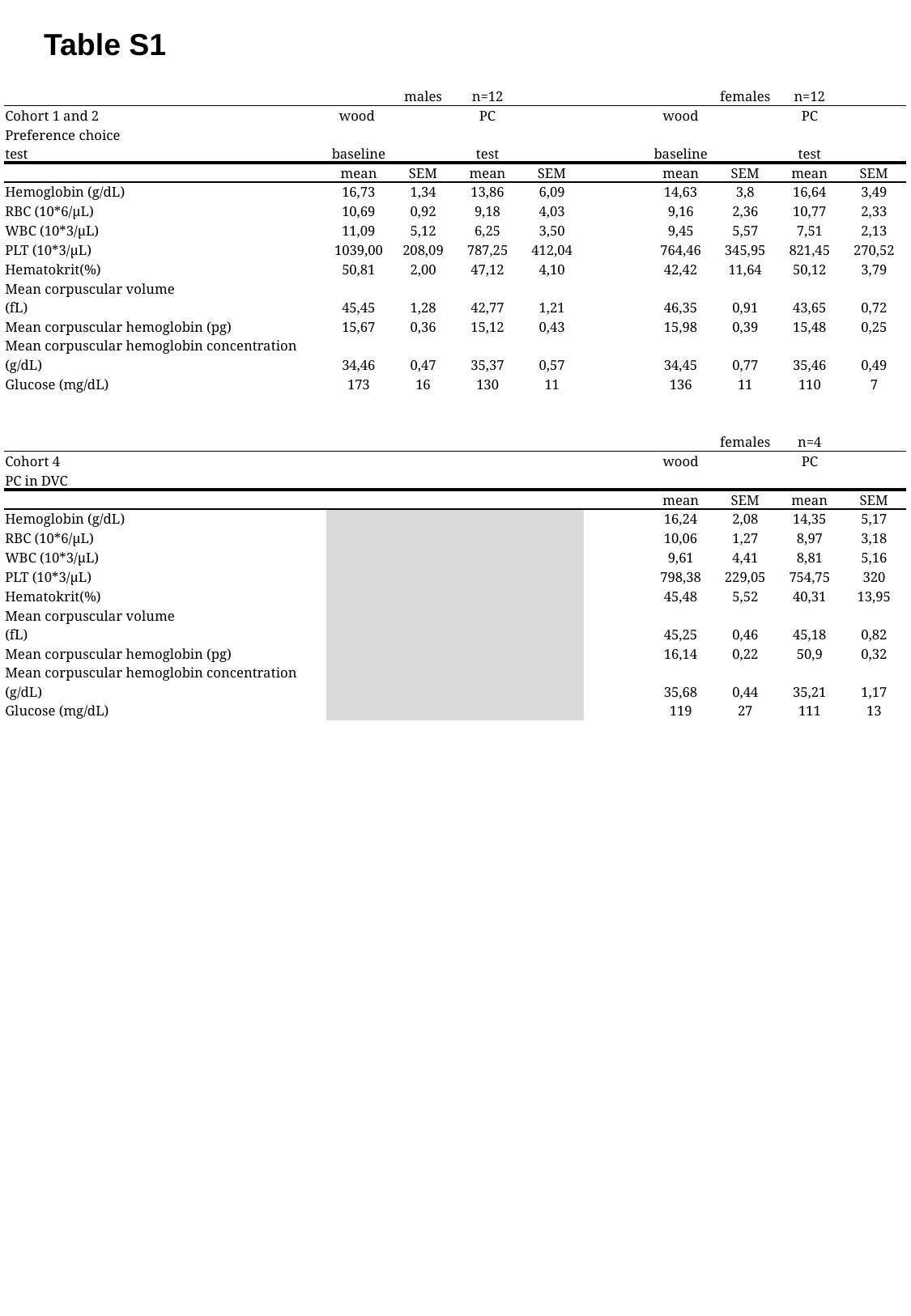

Table S1
| | | | | | | males | n=12 | | | | females | n=12 | |
| --- | --- | --- | --- | --- | --- | --- | --- | --- | --- | --- | --- | --- | --- |
| Cohort 1 and 2 | | | | | wood | | PC | | | wood | | PC | |
| Preference choice test | | | | | baseline | | test | | | baseline | | test | |
| | | | | | mean | SEM | mean | SEM | | mean | SEM | mean | SEM |
| Hemoglobin (g/dL) | | | | | 16,73 | 1,34 | 13,86 | 6,09 | | 14,63 | 3,8 | 16,64 | 3,49 |
| RBC (10\*6/µL) | | | | | 10,69 | 0,92 | 9,18 | 4,03 | | 9,16 | 2,36 | 10,77 | 2,33 |
| WBC (10\*3/µL) | | | | | 11,09 | 5,12 | 6,25 | 3,50 | | 9,45 | 5,57 | 7,51 | 2,13 |
| PLT (10\*3/µL) | | | | | 1039,00 | 208,09 | 787,25 | 412,04 | | 764,46 | 345,95 | 821,45 | 270,52 |
| Hematokrit(%) | | | | | 50,81 | 2,00 | 47,12 | 4,10 | | 42,42 | 11,64 | 50,12 | 3,79 |
| Mean corpuscular volume (fL) | | | | | 45,45 | 1,28 | 42,77 | 1,21 | | 46,35 | 0,91 | 43,65 | 0,72 |
| Mean corpuscular hemoglobin (pg) | | | | | 15,67 | 0,36 | 15,12 | 0,43 | | 15,98 | 0,39 | 15,48 | 0,25 |
| Mean corpuscular hemoglobin concentration (g/dL) | | | | | 34,46 | 0,47 | 35,37 | 0,57 | | 34,45 | 0,77 | 35,46 | 0,49 |
| Glucose (mg/dL) | | | | | 173 | 16 | 130 | 11 | | 136 | 11 | 110 | 7 |
| | | | | | | | | | | | | | |
| | | | | | | | | | | | | | |
| | | | | | | | | | | | females | n=4 | |
| Cohort 4 | | | | | | | | | | wood | | PC | |
| PC in DVC | | | | | | | | | | | | | |
| | | | | | | | | | | mean | SEM | mean | SEM |
| Hemoglobin (g/dL) | | | | | | | | | | 16,24 | 2,08 | 14,35 | 5,17 |
| RBC (10\*6/µL) | | | | | | | | | | 10,06 | 1,27 | 8,97 | 3,18 |
| WBC (10\*3/µL) | | | | | | | | | | 9,61 | 4,41 | 8,81 | 5,16 |
| PLT (10\*3/µL) | | | | | | | | | | 798,38 | 229,05 | 754,75 | 320 |
| Hematokrit(%) | | | | | | | | | | 45,48 | 5,52 | 40,31 | 13,95 |
| Mean corpuscular volume (fL) | | | | | | | | | | 45,25 | 0,46 | 45,18 | 0,82 |
| Mean corpuscular hemoglobin (pg) | | | | | | | | | | 16,14 | 0,22 | 50,9 | 0,32 |
| Mean corpuscular hemoglobin concentration (g/dL) | | | | | | | | | | 35,68 | 0,44 | 35,21 | 1,17 |
| Glucose (mg/dL) | | | | | | | | | | 119 | 27 | 111 | 13 |

## Slide 3
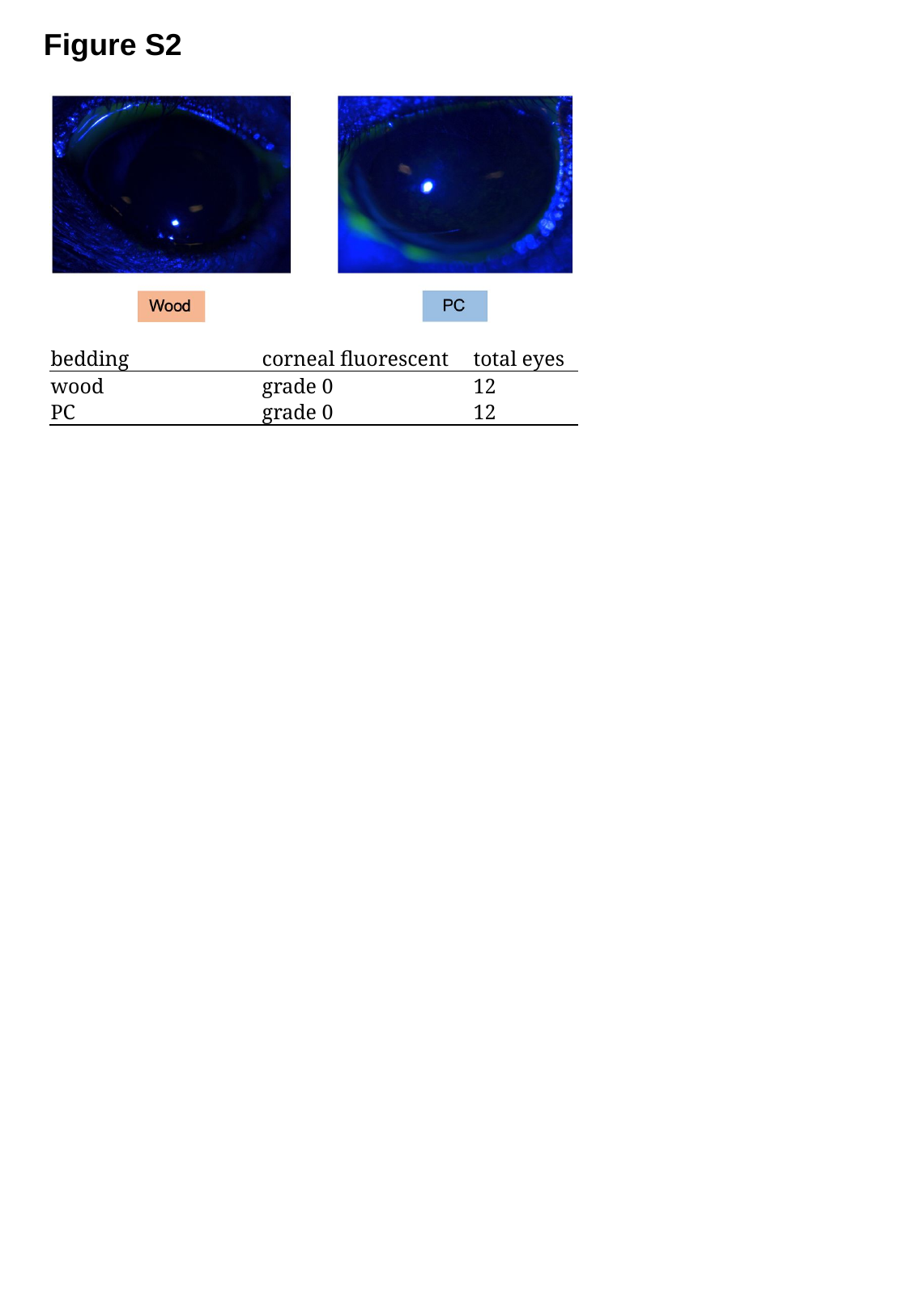

Figure S2
| bedding | | corneal fluorescent | | total eyes |
| --- | --- | --- | --- | --- |
| wood | | grade 0 | | 12 |
| PC | | grade 0 | | 12 |

## Slide 4
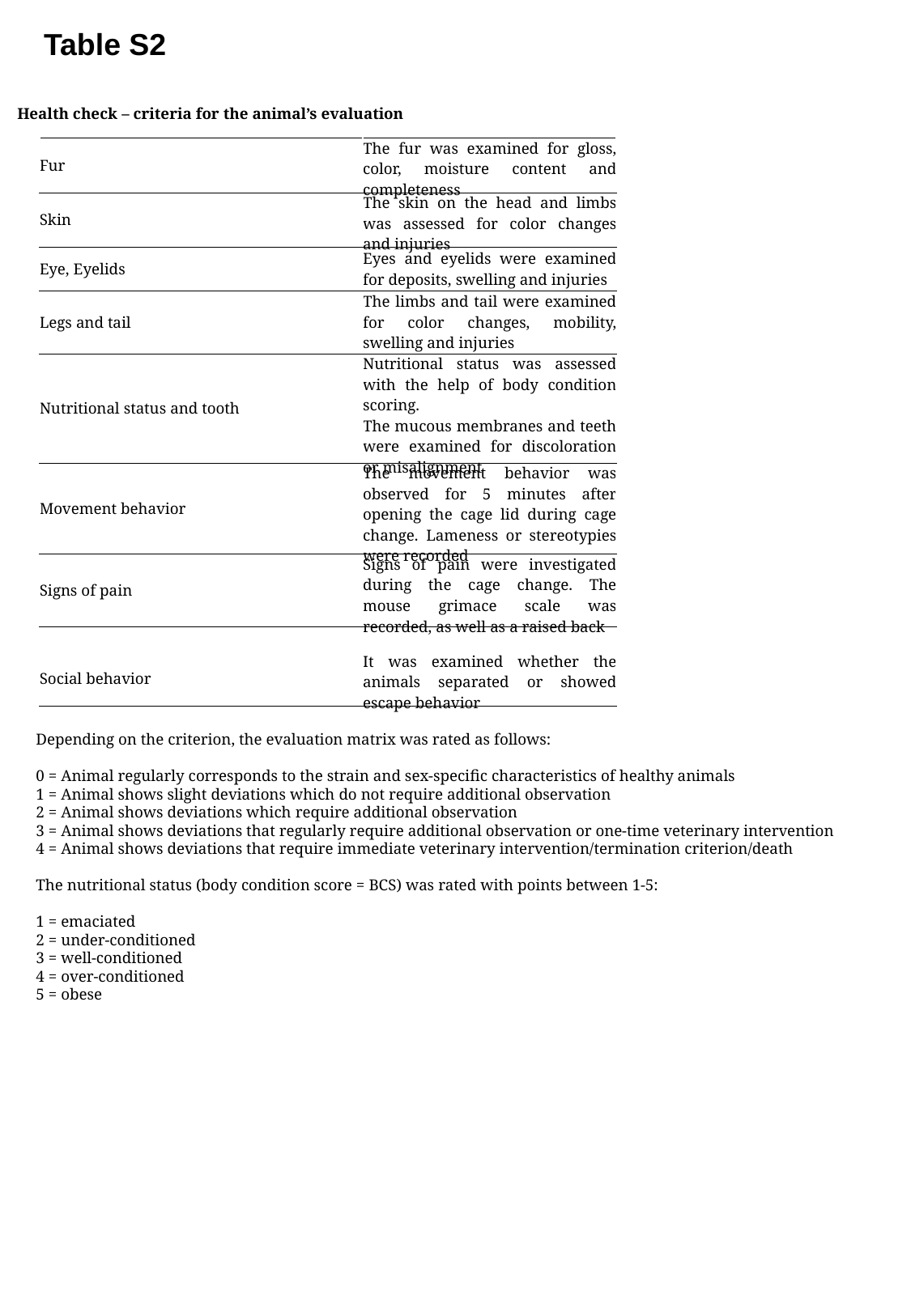

Table S2
Health check – criteria for the animal’s evaluation
| Fur | The fur was examined for gloss, color, moisture content and completeness |
| --- | --- |
| Skin | The skin on the head and limbs was assessed for color changes and injuries |
| Eye, Eyelids | Eyes and eyelids were examined for deposits, swelling and injuries |
| Legs and tail | The limbs and tail were examined for color changes, mobility, swelling and injuries |
| Nutritional status and tooth | Nutritional status was assessed with the help of body condition scoring. The mucous membranes and teeth were examined for discoloration or misalignment |
| Movement behavior | The movement behavior was observed for 5 minutes after opening the cage lid during cage change. Lameness or stereotypies were recorded |
| Signs of pain | Signs of pain were investigated during the cage change. The mouse grimace scale was recorded, as well as a raised back |
| | |
| Social behavior | It was examined whether the animals separated or showed escape behavior |
Depending on the criterion, the evaluation matrix was rated as follows:
0 = Animal regularly corresponds to the strain and sex-specific characteristics of healthy animals
1 = Animal shows slight deviations which do not require additional observation
2 = Animal shows deviations which require additional observation
3 = Animal shows deviations that regularly require additional observation or one-time veterinary intervention
4 = Animal shows deviations that require immediate veterinary intervention/termination criterion/death
The nutritional status (body condition score = BCS) was rated with points between 1-5:
1 = emaciated
2 = under-conditioned
3 = well-conditioned
4 = over-conditioned
5 = obese

## Slide 5
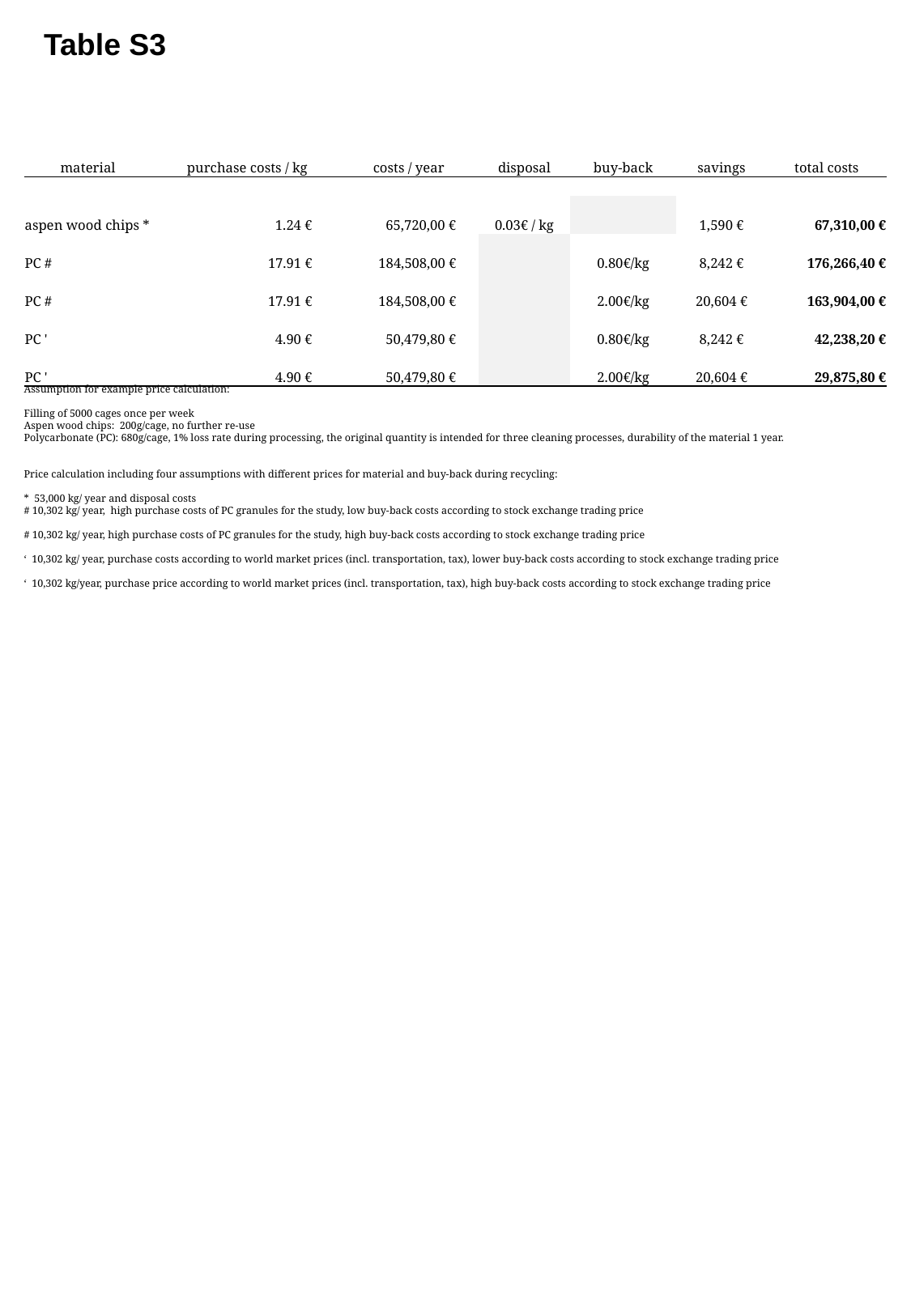

Table S3
| material | purchase costs / kg | costs / year | disposal | buy-back | savings | total costs |
| --- | --- | --- | --- | --- | --- | --- |
| | | | | | | |
| aspen wood chips \* | 1.24 € | 65,720,00 € | 0.03€ / kg | | 1,590 € | 67,310,00 € |
| PC # | 17.91 € | 184,508,00 € | | 0.80€/kg | 8,242 € | 176,266,40 € |
| PC # | 17.91 € | 184,508,00 € | | 2.00€/kg | 20,604 € | 163,904,00 € |
| PC ' | 4.90 € | 50,479,80 € | | 0.80€/kg | 8,242 € | 42,238,20 € |
| PC ' | 4.90 € | 50,479,80 € | | 2.00€/kg | 20,604 € | 29,875,80 € |
Assumption for example price calculation:
Filling of 5000 cages once per week
Aspen wood chips: 200g/cage, no further re-use
Polycarbonate (PC): 680g/cage, 1% loss rate during processing, the original quantity is intended for three cleaning processes, durability of the material 1 year.
Price calculation including four assumptions with different prices for material and buy-back during recycling:
* 53,000 kg/ year and disposal costs
# 10,302 kg/ year, high purchase costs of PC granules for the study, low buy-back costs according to stock exchange trading price
# 10,302 kg/ year, high purchase costs of PC granules for the study, high buy-back costs according to stock exchange trading price
‘ 10,302 kg/ year, purchase costs according to world market prices (incl. transportation, tax), lower buy-back costs according to stock exchange trading price
‘ 10,302 kg/year, purchase price according to world market prices (incl. transportation, tax), high buy-back costs according to stock exchange trading price
